# Supplementary material for: Identification of an embryonic differentiation stage marked by Sox1 and FoxA2 co-expression using combined cell tracking and high dimensional protein imaging
Source: Nat Commun. 2024 Sep 9;15:7860. doi: 10.1038/s41467-024-52069-z (PMC11385471; doi:10.1038/s41467-024-52069-z)
Supplement: Supplementary file 1 — Supplementary Information [file 41467_2024_52069_MOESM1_ESM.pdf]

Supp Fig. 1

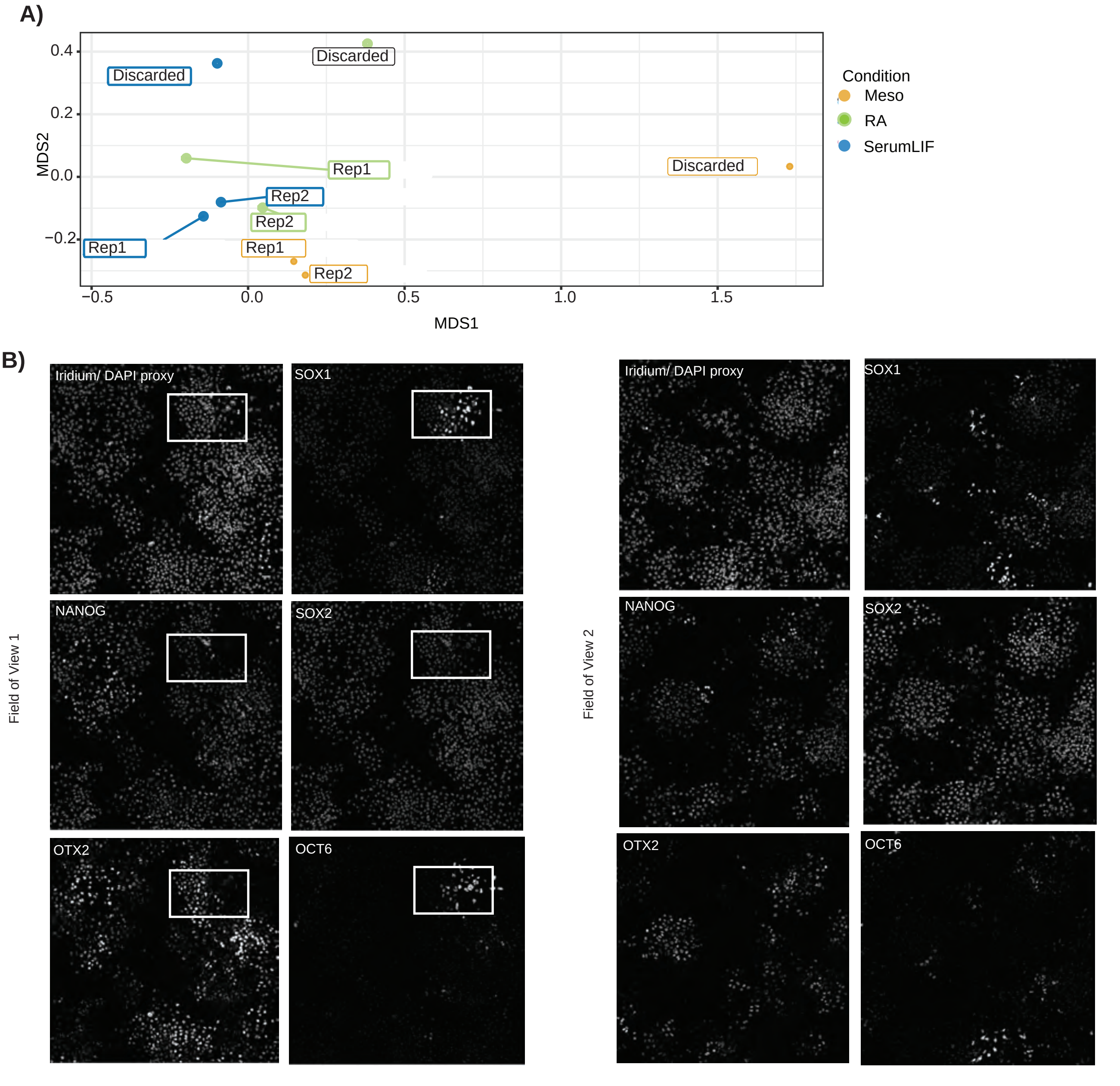
A)
Discarded
Discarded
Discarded
Rep1
Rep2
Rep2
Rep1
Rep1
Rep2
MDS2
0.4
0.2
0.0
−0.2
−0.5
0.0
0.5
1.0
1.5
MDS1
Condition
Meso
RA
SerumLIF
B)
Iridium/ DAPI proxy
SOX1
NANOG
SOX2
OTX2
OCT6
Field of View 1
Iridium/ DAPI proxy
SOX1
NANOG
SOX2
OTX2
OCT6
Field of View 2

Supplementary Fig. 1 Multidimensional protein expression from Imaging Mass Cytometry reveals heterogeneous protein expression between single cells within same media

A) *Multidimensional scaling plot (MDS) based on protein expression reveals that two replicates (Rep1, Rep2) are similar with no batch effects. An experiment using the same antibodies but where the cells detached partially prior to image acquisition (Discarded) also plotted to visualize degree of similarity between the two biological replicates.*

B) *Representative images in RA post IMC of two complete fields of view reveal heterogeneous protein expression at single cell level. Boxed area (white rectangle) in Field of View 1 is the zoomed in colony shown in Fig. 1B. n= 2 biological replicates. Scale bar 50 um.*

Supp Fig. 2

A)

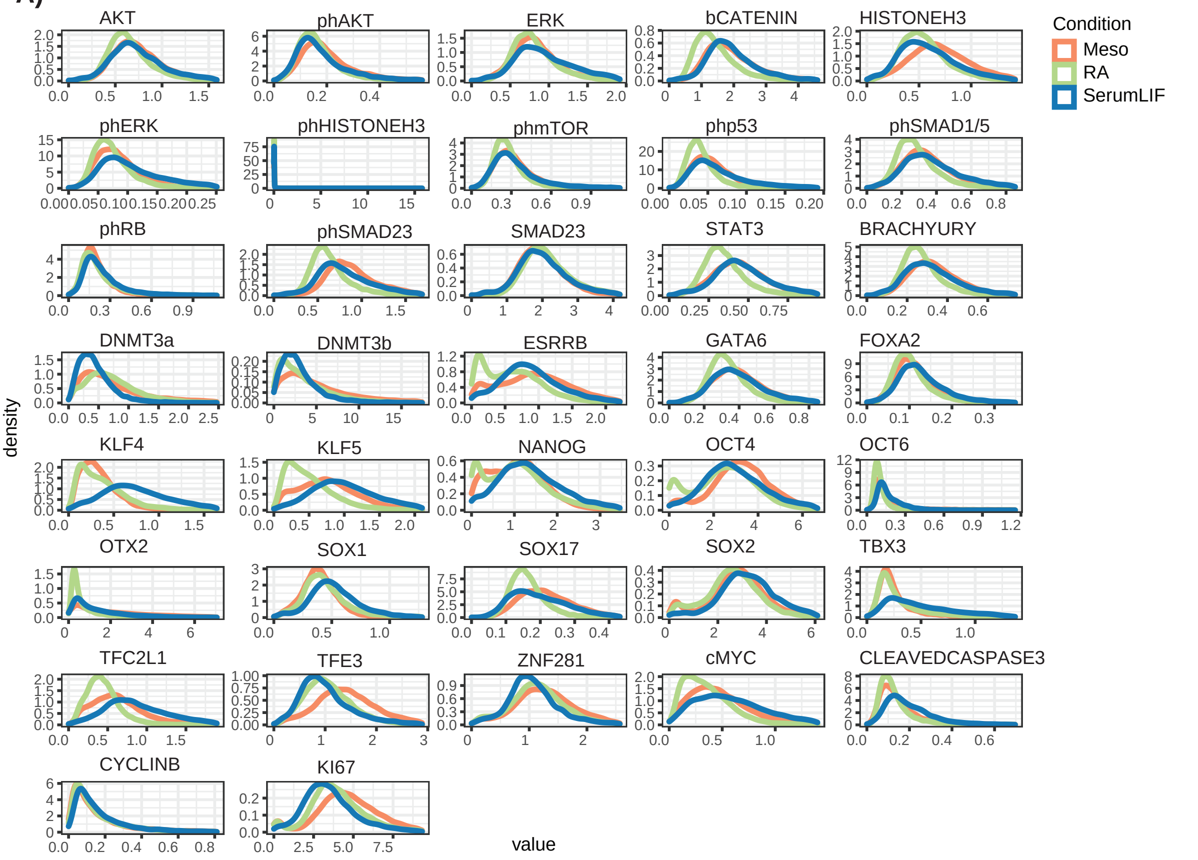
AKT
phAKT
ERK
bCATENIN
HISTONEH3
Condition
Meso
RA
SerumLIF
phERK
phHISTONEH3
phmTOR
php53
phSMAD1/5
phRB
phSMAD23
SMAD23
STAT3
BRACHYURY
DNMT3a
DNMT3b
ESRRB
GATA6
FOXA2
KLF4
KLF5
NANOG
OCT4
OCT6
OTX2
SOX1
SOX17
SOX2
TBX3
TFC2L1
TFE3
ZNF281
cMYC
CLEAVEDCASPASE3
CYCLINB
KI67
density
value

B)

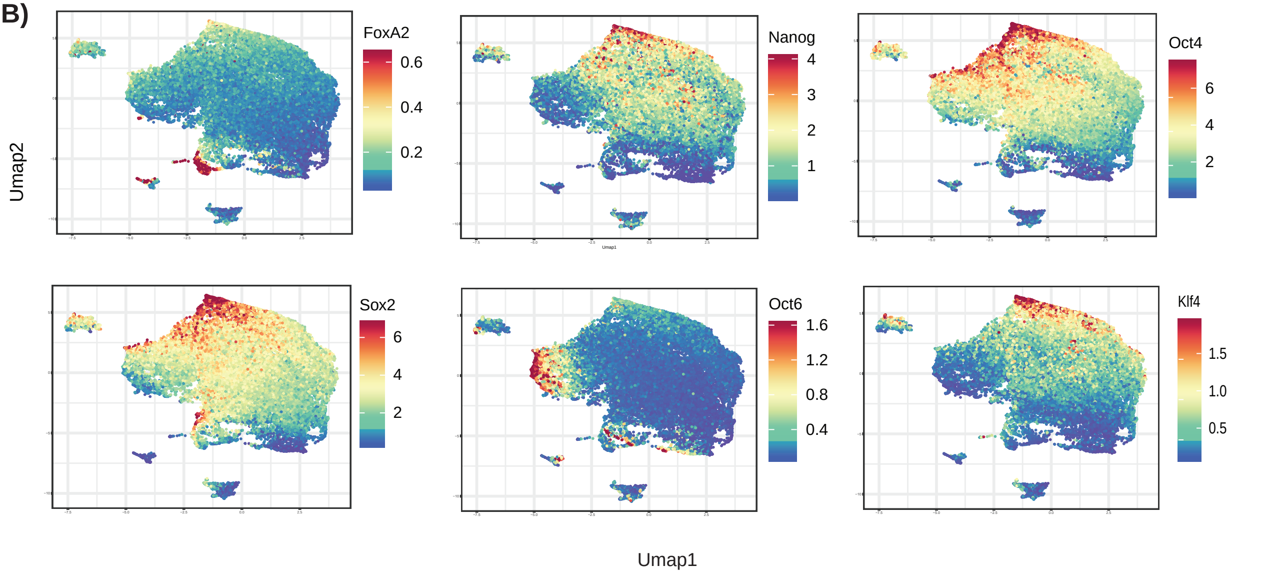
FoxA2
Nanog
Oct4
Sox2
Oct6
Klf4
Umap2
Umap1

Supplementary Fig. 2 Multidimensional protein expression from Imaging Mass Cytometry reveals heterogeneous protein expression distribution between cells in different media that cannot be resolved with umap analysis.

A) *Distribution of expression for all proteins in the IMC panel in different media. n>9000 cells per condition from two biological replicates*

B) *Umap based on protein expression from IMC and cell morphometric attributes as shown in Fig.1D, colored by various transcription factors' expression as indicated, fails to separate cells into clear distinct populations. n>34000 cells from two biological replicates.*

Supp Fig. 3

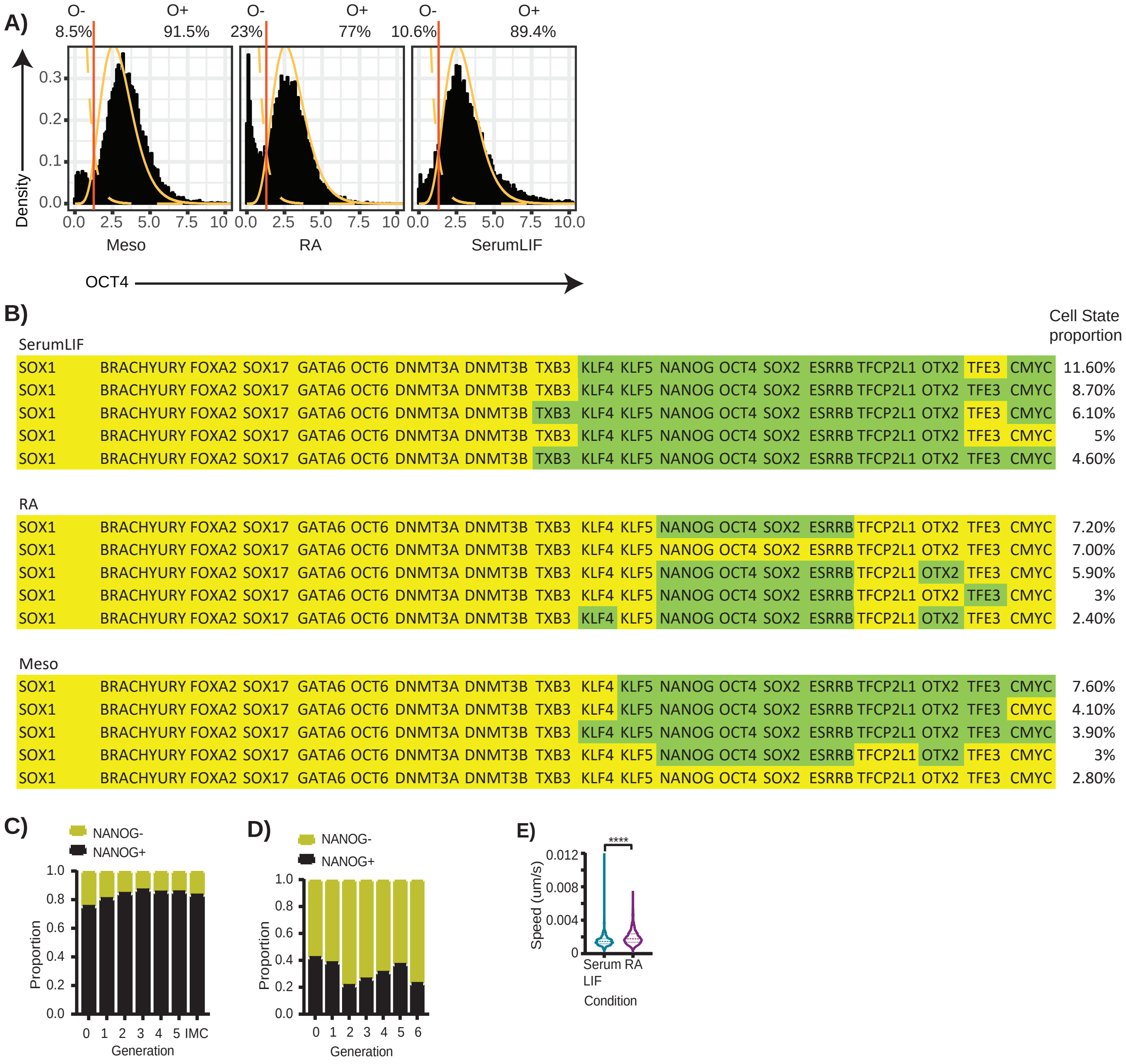
A)
O-
8.5%
O+
91.5%
O-
23%
O+
77%
O-
10.6%
O+
89.4%
Density
0.0
0.1
0.2
0.3
0.0 2.5 5.0 7.5 10
Meso
RA
SerumLIF
OCT4
B)
Cell State proportion
SerumLIF
SOX1 BRACHYURY FOXA2 SOX17 GATA6 OCT6 DNMT3A DNMT3B TXB3 KLF4 KLF5 NANOG OCT4 SOX2 ESRRB TFCP2L1 OTX2 TFE3 CMYC 11.60%
SOX1 BRACHYURY FOXA2 SOX17 GATA6 OCT6 DNMT3A DNMT3B TXB3 KLF4 KLF5 NANOG OCT4 SOX2 ESRRB TFCP2L1 OTX2 TFE3 CMYC 8.70%
SOX1 BRACHYURY FOXA2 SOX17 GATA6 OCT6 DNMT3A DNMT3B TXB3 KLF4 KLF5 NANOG OCT4 SOX2 ESRRB TFCP2L1 OTX2 TFE3 CMYC 6.10%
SOX1 BRACHYURY FOXA2 SOX17 GATA6 OCT6 DNMT3A DNMT3B TXB3 KLF4 KLF5 NANOG OCT4 SOX2 ESRRB TFCP2L1 OTX2 TFE3 CMYC 5%
SOX1 BRACHYURY FOXA2 SOX17 GATA6 OCT6 DNMT3A DNMT3B TXB3 KLF4 KLF5 NANOG OCT4 SOX2 ESRRB TFCP2L1 OTX2 TFE3 CMYC 4.60%
RA
SOX1 BRACHYURY FOXA2 SOX17 GATA6 OCT6 DNMT3A DNMT3B TXB3 KLF4 KLF5 NANOG OCT4 SOX2 ESRRB TFCP2L1 OTX2 TFE3 CMYC 7.20%
SOX1 BRACHYURY FOXA2 SOX17 GATA6 OCT6 DNMT3A DNMT3B TXB3 KLF4 KLF5 NANOG OCT4 SOX2 ESRRB TFCP2L1 OTX2 TFE3 CMYC 7.00%
SOX1 BRACHYURY FOXA2 SOX17 GATA6 OCT6 DNMT3A DNMT3B TXB3 KLF4 KLF5 NANOG OCT4 SOX2 ESRRB TFCP2L1 OTX2 TFE3 CMYC 5.90%
SOX1 BRACHYURY FOXA2 SOX17 GATA6 OCT6 DNMT3A DNMT3B TXB3 KLF4 KLF5 NANOG OCT4 SOX2 ESRRB TFCP2L1 OTX2 TFE3 CMYC 3%
SOX1 BRACHYURY FOXA2 SOX17 GATA6 OCT6 DNMT3A DNMT3B TXB3 KLF4 KLF5 NANOG OCT4 SOX2 ESRRB TFCP2L1 OTX2 TFE3 CMYC 2.40%
Meso
SOX1 BRACHYURY FOXA2 SOX17 GATA6 OCT6 DNMT3A DNMT3B TXB3 KLF4 KLF5 NANOG OCT4 SOX2 ESRRB TFCP2L1 OTX2 TFE3 CMYC 7.60%
SOX1 BRACHYURY FOXA2 SOX17 GATA6 OCT6 DNMT3A DNMT3B TXB3 KLF4 KLF5 NANOG OCT4 SOX2 ESRRB TFCP2L1 OTX2 TFE3 CMYC 4.10%
SOX1 BRACHYURY FOXA2 SOX17 GATA6 OCT6 DNMT3A DNMT3B TXB3 KLF4 KLF5 NANOG OCT4 SOX2 ESRRB TFCP2L1 OTX2 TFE3 CMYC 3.90%
SOX1 BRACHYURY FOXA2 SOX17 GATA6 OCT6 DNMT3A DNMT3B TXB3 KLF4 KLF5 NANOG OCT4 SOX2 ESRRB TFCP2L1 OTX2 TFE3 CMYC 3%
SOX1 BRACHYURY FOXA2 SOX17 GATA6 OCT6 DNMT3A DNMT3B TXB3 KLF4 KLF5 NANOG OCT4 SOX2 ESRRB TFCP2L1 OTX2 TFE3 CMYC 2.80%
C)
NANOG-
NANOG+
Proportion
0.0 0.2 0.4 0.6 0.8 1.0
0 1 2 3 4 5 IMC
Generation
D)
NANOG-
NANOG+
Proportion
0.0 0.2 0.4 0.6 0.8 1.0
0 1 2 3 4 5 6
Generation
E)
****
Speed (um/s)
0 0.004 0.008 0.012
Serum LIF
RA
Condition

Supplementary Fig. 3 Frequency of cell states per condition based on manual thresholding.

A) *Quantification of Oct4 antibody expression in different media conditions. Bimodal expression fitted with gamma distributions (yellow) and threshold value (orange) fixed at intersection of the two distributions to classify cells as Oct4+ (O+) or Oct4- (O-). Proportions of cells in each class per condition as indicated. n>9000 cells per condition from two biological replicates.*

B) *Top 5 cell states and their proportion of the population per media condition based on manual thresholding per transcription factor in panel. Green indicates upregulated and yellow indicates downregulated factors. N>9000 cells per condition from two independent replicates.*

C) *Proportions of NANOG+/- cells remains constant across generations and is similar with manual (Fig. 2A) and IMC thresholding (Fig.1F) in SerumLIF. n> 600 data points (each timepoint of all cells) per column from 2 biological replicates.*

D) *NANOG is downregulated in RA already from generation 1. n> 900 data points per column from 2 biological replicates*

E) *Original graph of zoomed in Fig.2G with all data included. Cells in RA are more motile than in SerumLIF. Motility quantified from timelapse as mean distance travelled per timelapse imaging frame. N=913 SerumLIF and 1636 RA cells from 2 biological replicates. Dotted lines represent 25th, 50th and 75th percentile values. ****p<0.0001, two-tailed unpaired t-test.*

Supp Fig. 4

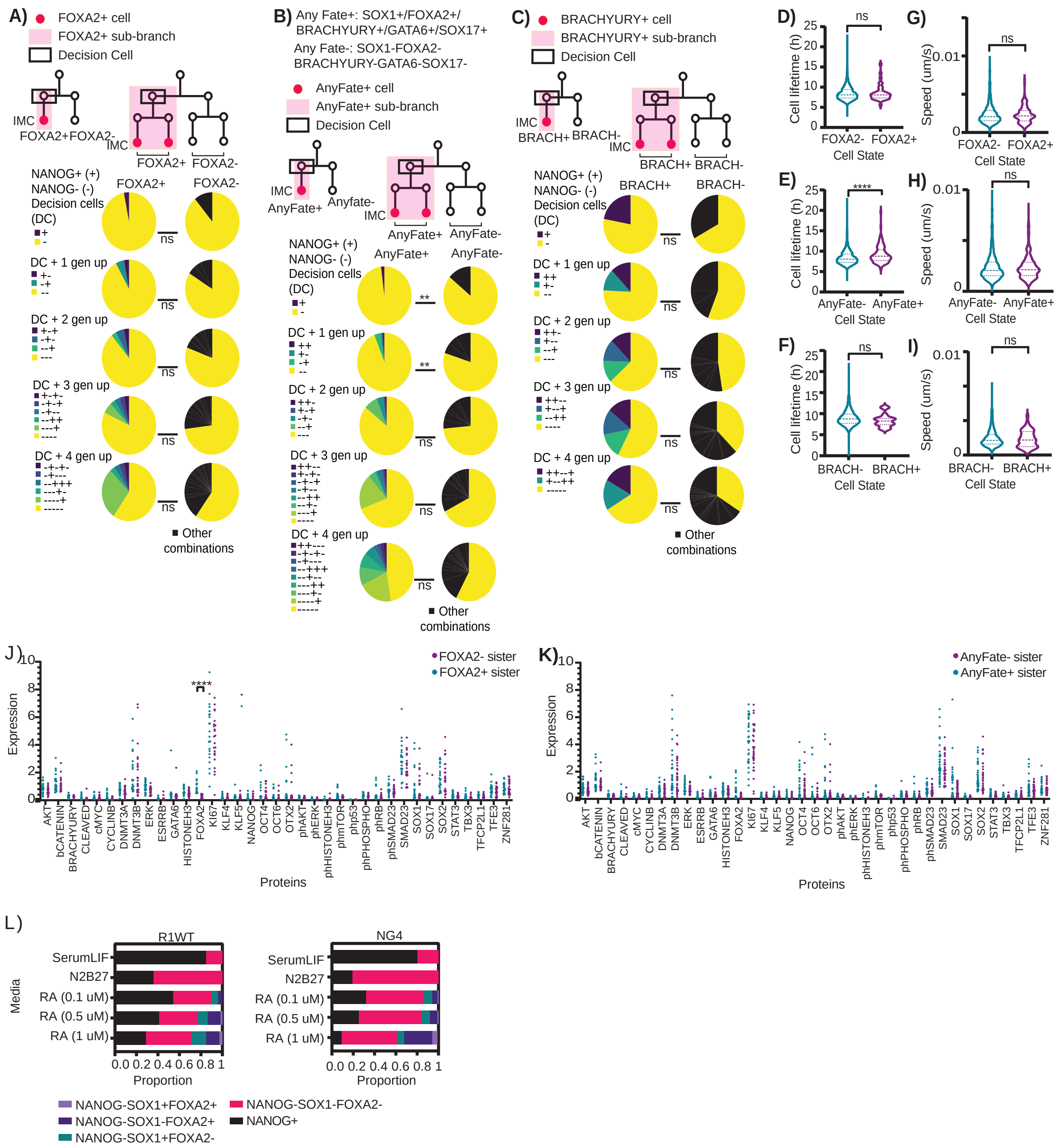

A) FOXA2+ cell
FOXA2+ sub-branch
Decision Cell
IMC
FOXA2+ FOXA2-
NANOG+ (+)
NANOG- (-)
Decision cells (DC)
DC + 1 gen up
DC + 2 gen up
DC + 3 gen up
DC + 4 gen up
ns
Other combinations
B) Any Fate+: SOX1+/FOXA2+/BRACHYURY+/GATA6+/SOX17+
Any Fate-: SOX1-FOXA2-BRACHYURY-GATA6-SOX17-
AnyFate+ cell
AnyFate+ sub-branch
Decision Cell
AnyFate+ Anyfate-
**
C) BRACHYURY+ cell
BRACHYURY+ sub-branch
Decision Cell
BRACH+ BRACH-
D) Cell lifetime (h)
Cell State
FOXA2- FOXA2+
E) ****
AnyFate- AnyFate+
F) BRACH- BRACH+
G) Speed (um/s)
H)
I)
J) Expression
Proteins
FOXA2- sister
FOXA2+ sister
AKT bCATENIN BRACHYURY CLEAVED cMYC CYCLINB DNMT3A DNMT3B ERK ESRRB GATA6 HISTONEH3 FOXA2 KI67 KLF4 KLF5 NANOG OCT4 OCT6 OTX2 phAKT phERK phHISTONEH3 phmTOR php53 phPHOSPHO phRB phSMAD23 SMAD23 SOX1 SOX17 SOX2 STAT3 TBX3 TFCP2L1 TFE3 ZNF281
K) AnyFate- sister
AnyFate+ sister
L) R1WT
NG4
Media
SerumLIF
N2B27
RA (0.1 uM)
RA (0.5 uM)
RA (1 uM)
Proportion
NANOG-SOX1+FOXA2+
NANOG-SOX1-FOXA2+
NANOG-SOX1+FOXA2-
NANOG-SOX1-FOXA2-
NANOG+

Supplementary Fig. 4 Nanog dynamics, cell motility and lifetime are not indicators of germ layer marker upregulation.

A-C) *NANOG downregulation occurs two generations prior to but is not sufficient for germ layer marker upregulation as determined by Decision Cells. Ancestral NANOG levels of Fate+ Decision Cells (left) and Fate- cells (right) as indicated. Decision Cells, i.e. ancestor cells in which a lineage marker is first expressed, (in rectangle box) detected as shown graphically (see Methods). Cells classified as NANOG+ (+) or NANOG- (-) based on NANOG reporter expression above or below threshold value for ~2.5 hours. "+-" means current cell is NANOG+ and its mother cell is NANOG-, and so on. Two-sided Fisher's exact test to determine if observed proportions are significantly different, with cells in yellow as category 1 and all other combinations grouped together under category 2.* ***A)*** *n=39 FOXA2+ Decision Cells and n=359 FOXA2- cells from two biological replicates. ns: not significant p=0.1, 0.23, 0.19, 0.23, 0.84 (DC,1,2,3,4 gen up)* ***B)*** *n= 84 AnyFate+ Decision Cells and n=239 AnyFate- cells from two biological replicates. **p=0.002, 0.004 (DC,1 gen up) ns: not significant p=0.06, 0.88, 0.26 (2,3,4 gen up)* ***C)*** *n= 9 Brachyury+ Decision Cells and n = 318 Brachyury- cells from two biological replicates. ns: not significant p=0.72, 0.47, 0.48, 0.43, 0.18 (DC, 1,2,3,4 gen up).*

D-F) *Cell lifetime is not a reliable marker of germ layer marker expression. Dotted lines represent 25th, 50th and 75th percentile values, Two-tailed unpaired t-test.* ***D)*** *n=36 FOXA2+ and n=1533 FOXA2- cells from 2 biological replicates. ns: not significant p=0.96.* ***E)*** *AnyFate+ and AnyFate- cells in RA have similar lifetimes (effect size: 45 minutes). N=169 AnyFate+ and n=1400 AnyFate- cells from 2 biological replicates. ****p=* 1.38E-06*, Cannot be used as a predictive indicator of germ layer marker expression.* ***F)*** *n=16 Brachyury+ and n=2002 Brachyury- cells from 2 biological replicates. ns: not significant p=0.22*

G-I) *Cell speed is not a marker of germ layer marker expression. Dotted lines represent 25th, 50th and 75th percentile values. Two-tailed unpaired t-test.* ***G)*** *n=119 FOXA2+ and n=3184 FOXA2- cells from 2 biological replicates ns: not significant p=0.52* ***H)*** *n=449 AnyFate+ and n=2854 AnyFate- cells from 2 biological replicates. ns: not significant p=0.61* ***I)*** *n=46 BRACHYURY+ and n=4206 BRACHYURY- cells from 2 biological replicates. ns: not significant p=0.56*

J) *No significant differences in pluripotency marker and signaling molecule expression of FOXA2+/FOXA2- sister pairs. N=23 sister pairs from two biological replicates. ****p=* 0.000002 *where indicated, else non-significant p>0.2, unpaired t tests corrected for multiple comparisons.*

K) *No significant differences in pluripotency marker and signaling molecule expression of AnyFate+/AnyFate- sister pairs. N=34 sister pairs from two biological replicates. All non-significant p>0.1, unpaired t tests corrected for multiple comparisons.*

L) *SOX1+FOXA2+ cells are generated in different RA concentrations. Proportion of cells in R1WT (left) and NG4 (right) ESC lines. n>700 cells per condition per cell line from 1 biological replicate each.*

Supp Fig. 5

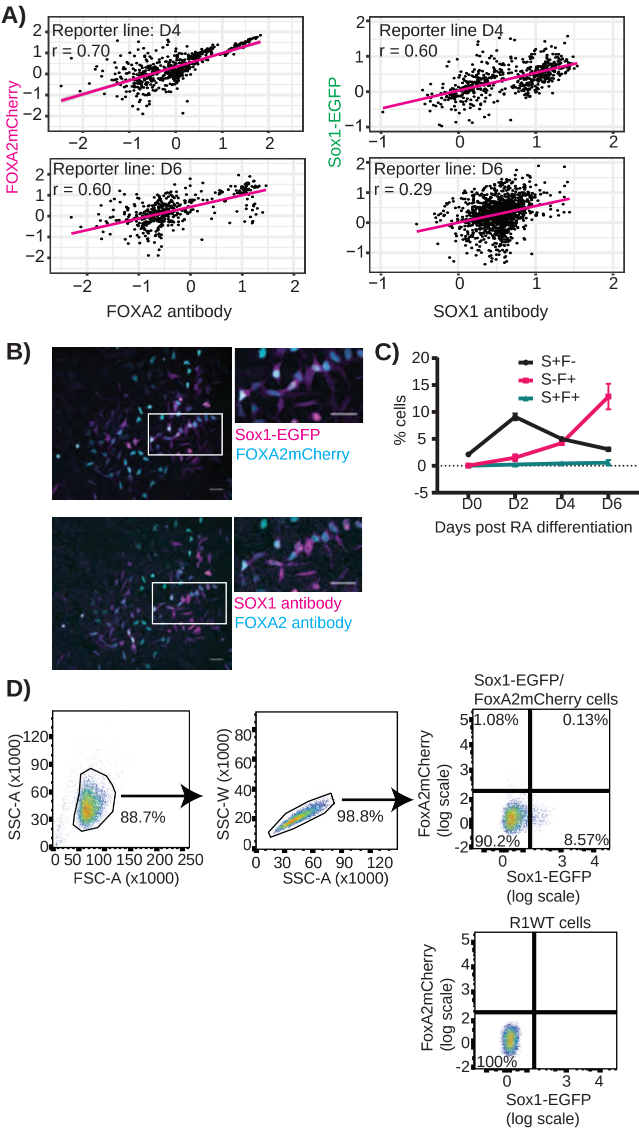
A)
Reporter line: D4
r = 0.70
Reporter line: D6
r = 0.60
FOXA2mCherry
FOXA2 antibody
Reporter line D4
r = 0.60
Reporter line: D6
r = 0.29
Sox1-EGFP
SOX1 antibody
B)
Sox1-EGFP
FOXA2mCherry
SOX1 antibody
FOXA2 antibody
C)
S+F-
S-F+
S+F+
% cells
D0
D2
D4
D6
Days post RA differentiation
D)
SSC-A (x1000)
FSC-A (x1000)
88.7%
SSC-W (x1000)
SSC-A (x1000)
98.8%
Sox1-EGFP/
FoxA2mCherry cells
1.08%
0.13%
90.2%
8.57%
FoxA2mCherry (log scale)
Sox1-EGFP (log scale)
R1WT cells
100%

Supplementary Fig. 5 FoxA2mCherry reports Foxa2 protein levels accurately.

A) *The Sox-EGFP/FoxA2mCherry line accurately reports FOXA2 at 4- and 6-days post RA differentiation and SOX1 protein expression till D4 post RA differentiation. Linear regression line (pink) and Pearson correlation coefficient (r) as indicated. Data log transformed. n>300 cells per day from two biological replicates.*

B) *Overlay images of gray scale images from Fig. 4C. Scale bar 50 um*

C) *Proportion of Sox1+FOXA2- cells peaks at 2 days post RA differentiation while proportion of Sox1-FOXA2+ cells increase steadily. Sox1+FOXA2+ cells remain a minor but persistent population. Data from FACS analysis of differentiating Sox1-EGFP/FoxA2mCherry cells. n= 3 biological replicates per data point. Error bars represent means ± SDs.*

D) *FACS gating strategy to identify relevant populations shown in C). R1WT cells as negative control to set fluorescence thresholds.*

Supp Fig. 6

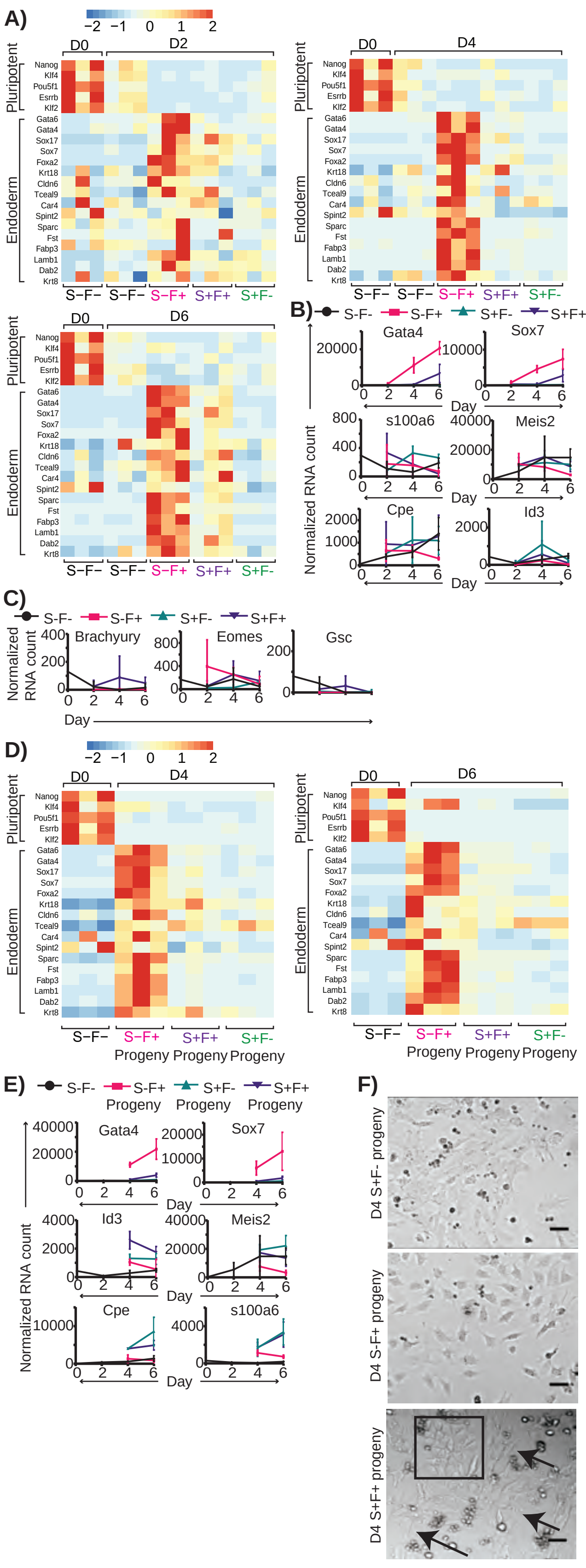
A)
-2 -1 0 1 2
D0
D2
D4
D6
Pluripotent
Endoderm
Nanog
Klf4
Pou5f1
Esrrb
Klf2
Gata6
Gata4
Sox17
Sox7
Foxa2
Krt18
Cldn6
Tceal9
Car4
Spint2
Sparc
Fst
Fabp3
Lamb1
Dab2
Krt8
S−F− S−F− S−F+ S+F+ S+F-
B)
S-F- S-F+ S+F- S+F+
Gata4
Sox7
s100a6
Meis2
Cpe
Id3
Day
Normalized RNA count
C)
S-F- S-F+ S+F- S+F+
Brachyury
Eomes
Gsc
Normalized RNA count
Day
D)
-2 -1 0 1 2
D0
D4
D6
Pluripotent
Endoderm
S−F− S−F+ S+F+ S+F-
Progeny Progeny Progeny
E)
S-F- S-F+ S+F- S+F+
Progeny Progeny Progeny
Gata4
Sox7
Id3
Meis2
Cpe
s100a6
Day
Normalized RNA count
F)
D4 S+F- progeny
D4 S-F+ progeny
D4 S+F+ progeny

Supplementary Fig. 6: Visceral and parietal endoderm markers are upregulated at intermediate levels in Sox1+FOXA2+ cells and its progeny compared to Sox1-FOXA2+ cells and its progeny.

A) *Additional markers for visceral and parietal endoderm markers are also upregulated in Sox1-FOXA2+ cells and this signature becomes more pronounced with time. Sox1+FOXA2+ cells show intermediate expression for same markers. Relative gene expression per population as indicated, following z-score transformation of normalized counts by row.*

B) *Sox1+FOXA2+ cells express key endoderm and neuroectoderm markers in intermediate range over time. n= 3 biological replicates per condition. Error bars represent means ± SDs.*

C) *Markers for mesoendoderm, a precursor to definitive endoderm formation, are not expressed during RA differentiation. n= 3 biological replicates per condition. Error bars represent means ± SDs.*

D) *Additional markers for visceral and parietal endoderm are also upregulated in the progeny of Sox1-FOXA2+ cells. Sox1+FOXA2+ progeny population shows intermediate expression for same markers. Relative gene expression per population as indicated, following z-score transformation of normalized counts by row.*

E) *Progeny population of Sox1+FOXA2+ cells express both endoderm and neuroectoderm markers over time. n= 3 biological replicates per condition. Error bars represent means ± SDs.*

F) *Sox1+FOXA2+ cells give rise to cells with two distinct morphologies: flat, elongated characteristic of neuronal cells (arrows, D4 S+F- progeny) versus epithelial-like morphology (rectangle, D4 S-F+ progeny) characteristic of endoderm cells. N =2 biological replicates per condition. Scale bar 50 um.*

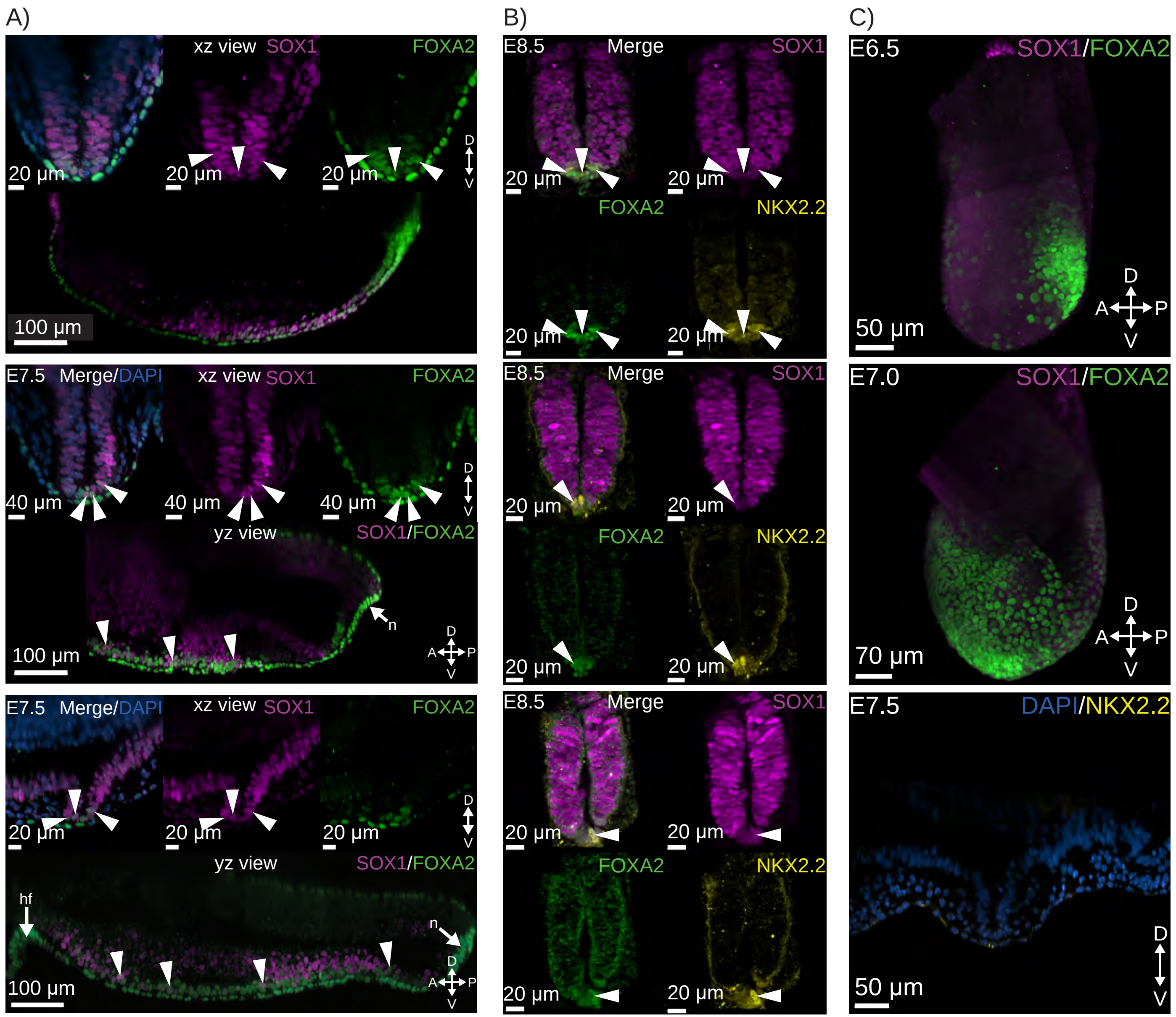

A)
xz view
SOX1
FOXA2
20 µm
D
V
100 µm
E7.5
Merge/DAPI
40 µm
yz view
SOX1/FOXA2
n
A
P
hf
B)
E8.5
Merge
NKX2.2
C)
E6.5
50 µm
E7.0
70 µm
DAPI/NKX2.2

Supplementary Fig.7 Immunofluorescence staining replicates of whole 7.5 embryos and E8.5 embryo from Fig. 6.

A) *Three E7.5 Embryos stained for SOX1 and FOXA2. SOX1+FOXA2+ cells were detected ventrally in the neural groove and along its whole length from posterior to anterior (marked with arrowheads. A: anterior, D: distal, P: posterior, V: ventral. hf: head fold, n: node, ng: neural groove, ve: visceral endoderm.*

B) *Neural tube slices from three E8.5 embryos, stained for SOX1, FOXA2, and the marker for p3 neural progenitors, NKX2.2. SOX1+FOXA2+ cells were still present at E8.5. The cells were located ventrally, and some were found to co-express NKX2.2, thus suggesting that SOX1+FOXA2+ cells could be precursors to p3 neural progenitors.*

C) *E6.5-7.5 embryos stained against SOX1 and FOXA2, and optical section and zoom in the neural groove of an E7.5 embryo, stained against NKX2.2. FOXA2 expression was detected both at E6.5 and E7.0, but no SOX1 expression was detected. No NKX2.2 expression was detected yet at E7.5.*

Target	Company	Catalogue number	Annotation	Isotope/Metal tag	Concentration
AKT	Cell Signaling Tech	C67E7	Signaling protein	Dy164	5 ug/ml
phAKT	Cell Signaling Tech	4060	Signaling protein	Eu153	6 ug/ml
BRACHYURY	R&D	AF2085	Transcription factor	Nd146	5 ug/ml
CLEAVED CASPASE	BD Biosciences	C92-605	Apoptosis marker	Ln115	4 ug/ml
CYCLIN B1	ThermoFisher	GNS11	Cell cycle marker	Gd156	3 ug/ml
DNMT3a	NovusBio	64B814.1	Histone modifier	Nd150	2 ug/ml
DNMT3b	NovusBio	52A1018	Histone modifier	Yb176	1 ug/ml
ESRRB	R&D	PP-H6705-00	Transcription factor	Yb174	1 ug/ml
ERK	Cell Signaling Tech	137F5	Signaling protein	Nd143	4 ug/ml
pERK	BD Biosciences	561991	Signaling protein	Sm154	5 ug/ml
GATA6	R&D	AF1700	Transcription factor	Nd142	4 ug/ml
FOXA2	Santa Cruz	6554	Transcription factor	Yb172	1 ug/ml
HISTONE H3	Cell Signaling Tech	4499	Histone modifier	Ln113	2 ug/ml
pHISTONE H3	Abcam	ab10543	Histone modifier	Er170	0.001 µg/ml
KLF4	Abcam	ab75486	Transcription factor	Gd155	2.5 ug/ml
KLF5	R&D	AF3758	Transcription factor	Sm152	2 ug/ml
KI67	Cell Signaling Tech	9449	Cell cycle marker	Er168	2 ug/ml
NANOG	eBio	MLC-51	Transcription factor	Gd160	2 ug/ml
OCT6	Millipore	MABN738	Transcription factor	Yb173	2.5 ug/ml
OCT4	Santa Cruz	sc8628	Transcription factor	Dy162	0.75 ug/ml
OTX2	R&D	AF1979	Transcription factor	Lu175	1 ug/ml
pSMAD1/5	Invitrogen	700047	Signaling protein	Nd144	2 ug/ml
pRB	Cell Signaling Tech	8516	Cell cycle marker	Nd148	2 ug/ml
SOX1	R&D	AF3369	Transcription factor	Dy161	1.5 ug/ml
SMAD2/3	Cell Signaling Tech	8685	Signaling protein	Tb159	2.5 ug/ml
pSMAD2/3	Cell Signaling Tech	8828	Signaling protein	Tm169	5 ug/ml
SOX17	Neuromics	GT15094	Transcription factor	Pr141	2 ug/ml
SOX2	Sigma-Aldrich	ab5603	Transcription factor	Eu151	2.5 ug/ml
STAT3	Cell Signaling Tech	9139	Signaling protein	Er167	5 ug/ml
TBX3	Santa Cruz	sc17871	Transcription factor	Yb171	1.5 ug/ml
TFE3	Sigma-Aldrich	HPA023881	Transcription factor	Nd145	2 ug/ml
TFCP2L1	R&D	AF5726	Transcription factor	Sm147	5 ug/ml
ZNF281	Santa Cruz	sc-166933	Transcription factor	Dy163	1.5 ug/ml
bCATENIN	Cell Signaling Tech	8814	Signaling protein	Ho165	2.5 ug/ml
cMYC	Cell Signaling Tech	5605	Cell cycle marker	Er166	2.5 ug/ml
pmTOR	Cell Signaling Tech	5536	Signaling protein	Gd158	5 ug/ml
php53	Cell Signaling Tech	9286	Signaling protein	Sm149	5 ug/ml

Supplementary Table 1 Antibody panel used in Imaging Mass Cytometry
