# Supplementary material for: Identification of an embryonic differentiation stage marked by Sox1 and FoxA2 co-expression using combined cell tracking and high dimensional protein imaging
Source: Nat Commun. 2024 Sep 9;15:7860. doi: 10.1038/s41467-024-52069-z (PMC11385471; doi:10.1038/s41467-024-52069-z)
Supplement: Supplementary file 3 — Description Of Additional Supplementary Files [file 41467_2024_52069_MOESM3_ESM.pdf]

Description of Additional supplementary files

Supplementary Movie.1 FOXA2 (green) and SOX1 (pink) immunostaining in E7.5 whole embryo to visualize SOX1+FOXA2+ cells in the ventral region of the neural groove.
