# Supplementary material for: Identification of an embryonic differentiation stage marked by Sox1 and FoxA2 co-expression using combined cell tracking and high dimensional protein imaging
Source: Nat Commun. 2024 Sep 9;15:7860. doi: 10.1038/s41467-024-52069-z (PMC11385471; doi:10.1038/s41467-024-52069-z)
Supplement: Supplementary file 5 — Reporting summary [file 41467_2024_52069_MOESM5_ESM.pdf]

nature portfolio

Corresponding author(s): Timm Schroeder

Last updated by author(s): Aug 16, 2024

Reporting Summary

Nature Portfolio wishes to improve the reproducibility of the work that we publish. This form provides structure for consistency and transparency in reporting. For further information on Nature Portfolio policies, see our Editorial Policies and the Editorial Policy Checklist.

Statistics

For all statistical analyses, confirm that the following items are present in the figure legend, table legend, main text, or Methods section.

n/a	Confirmed	
☐	☒	The exact sample size (*n*) for each experimental group/condition, given as a discrete number and unit of measurement
☐	☒	A statement on whether measurements were taken from distinct samples or whether the same sample was measured repeatedly
☐	☒	The statistical test(s) used AND whether they are one- or two-sided<br>*Only common tests should be described solely by name; describe more complex techniques in the Methods section.*
☒	☐	A description of all covariates tested
☐	☒	A description of any assumptions or corrections, such as tests of normality and adjustment for multiple comparisons
☐	☒	A full description of the statistical parameters including central tendency (e.g. means) or other basic estimates (e.g. regression coefficient) AND variation (e.g. standard deviation) or associated estimates of uncertainty (e.g. confidence intervals)
☐	☒	For null hypothesis testing, the test statistic (e.g. F, t, r) with confidence intervals, effect sizes, degrees of freedom and P value noted<br>*Give P values as exact values whenever suitable.*
☒	☐	For Bayesian analysis, information on the choice of priors and Markov chain Monte Carlo settings
☒	☐	For hierarchical and complex designs, identification of the appropriate level for tests and full reporting of outcomes
☐	☒	Estimates of effect sizes (e.g. Cohen's *d*, Pearson's *r*), indicating how they were calculated

Our web collection on statistics for biologists contains articles on many of the points above.

Software and code

Policy information about availability of computer code

Data collection	Youscope v2.1 for timelapse imaging: Open Source (http://langmo.github.io/youscope/)<br>Hyperion Imaging System for Imaging Mass Cytometry: Commercial from Standard Biotools (https://www.standardbio.com/products/instruments/hyperion), BD FACS Aria III, BD FACS Diva v8, Leica SP8 confocal, Zeiss LSM980 microscope 2 Photon, Illumina NovaSeq 6000 for RNASeq
Data analysis	Publicly available: Histocat v1.0 (IMC data quantification), Ilastik v0.5 (image segmentation), BaSiC v1.1(Timelapse data background correction), The Tracking Tool v3.5(Tree generation from timelapse data), qTfy v1.1(Quantifying tree data from timelapse imaging), fastER v1.4 (Timelapse image segmentation), R v4.1.2 (data analysis), MATLAB R2017B (data analysis), GraphPad Prism v10.1.2, FlowJo v10, Imaris v10

For manuscripts utilizing custom algorithms or software that are central to the research but not yet described in published literature, software must be made available to editors and reviewers. We strongly encourage code deposition in a community repository (e.g. GitHub). See the Nature Portfolio guidelines for submitting code & software for further information.

Data

Policy information about availability of data

All manuscripts must include a data availability statement. This statement should provide the following information, where applicable:

- Accession codes, unique identifiers, or web links for publicly available datasets
- A description of any restrictions on data availability
- For clinical datasets or third party data, please ensure that the statement adheres to our policy

All original imaging data will provided by lead contact upon request.
Quantified imaging data and analysis scripts are uploaded to ETH Research Collection with DOI 10.3929/ethz-b-000688864 [https://doi.org/10.3929/ethz-b-000688864].
Bulk RNASeq data has been uploaded to the Gene Expression Omnibus (GEO) database under accession code GSE259317 [https://www.ncbi.nlm.nih.gov/geo/query/acc.cgi?acc=GSE259317]. Source data are provided with this paper.

Research involving human participants, their data, or biological material

Policy information about studies with human participants or human data. See also policy information about sex, gender (identity/presentation), and sexual orientation and race, ethnicity and racism.

Reporting on sex and gender	*Use the terms sex (biological attribute) and gender (shaped by social and cultural circumstances) carefully in order to avoid confusing both terms. Indicate if findings apply to only one sex or gender; describe whether sex and gender were considered in study design; whether sex and/or gender was determined based on self-reporting or assigned and methods used. Provide in the source data disaggregated sex and gender data, where this information has been collected, and if consent has been obtained for sharing of individual-level data; provide overall numbers in this Reporting Summary. Please state if this information has not been collected. Report sex- and gender-based analyses where performed, justify reasons for lack of sex- and gender-based analysis.*
Reporting on race, ethnicity, or other socially relevant groupings	*Please specify the socially constructed or socially relevant categorization variable(s) used in your manuscript and explain why they were used. Please note that such variables should not be used as proxies for other socially constructed/relevant variables (for example, race or ethnicity should not be used as a proxy for socioeconomic status). Provide clear definitions of the relevant terms used, how they were provided (by the participants/respondents, the researchers, or third parties), and the method(s) used to classify people into the different categories (e.g. self-report, census or administrative data, social media data, etc.) Please provide details about how you controlled for confounding variables in your analyses.*
Population characteristics	*Describe the covariate-relevant population characteristics of the human research participants (e.g. age, genotypic information, past and current diagnosis and treatment categories). If you filled out the behavioural & social sciences study design questions and have nothing to add here, write "See above."*
Recruitment	*Describe how participants were recruited. Outline any potential self-selection bias or other biases that may be present and how these are likely to impact results.*
Ethics oversight	*Identify the organization(s) that approved the study protocol.*

Note that full information on the approval of the study protocol must also be provided in the manuscript.

Field-specific reporting

Please select the one below that is the best fit for your research. If you are not sure, read the appropriate sections before making your selection.

☒ Life sciences ☐ Behavioural & social sciences ☐ Ecological, evolutionary & environmental sciences

For a reference copy of the document with all sections, see nature.com/documents/nr-reporting-summary-flat.pdf

Life sciences study design

All studies must disclose on these points even when the disclosure is negative.

Sample size	No statistical methods were used to determine sample sizes
Data exclusions	In timelapse data, for certain analysis involving NANOG live reporter, the first two and last two timepoints of a cell were excluded to remove Nanog quantification pre-/post-mitosis when nuclear envelope breaks. This was pre-determined and also reported in Methods.
Replication	All experiments were replicated atleast twice. Replication was successful
Randomization	Samples were not randomized
Blinding	Investigators were not blinded during data collection/analysis

Reporting for specific materials, systems and methods

We require information from authors about some types of materials, experimental systems and methods used in many studies. Here, indicate whether each material, system or method listed is relevant to your study. If you are not sure if a list item applies to your research, read the appropriate section before selecting a response.

Materials & experimental systems

n/a	Involved in the study
☐	☒ Antibodies
☐	☒ Eukaryotic cell lines
☒	☐ Palaeontology and archaeology
☐	☒ Animals and other organisms
☒	☐ Clinical data
☒	☐ Dual use research of concern
☒	☐ Plants

Methods

n/a	Involved in the study
☒	☐ ChIP-seq
☐	☒ Flow cytometry
☒	☐ MRI-based neuroimaging

Antibodies

Antibodies used

Antibody Company CatalogueNumber Working Concentration

AKT Cell Signaling Tech C67E7 5ug/ml
phAKT Cell Signaling Tech 4060 6 ug/ml
BRACHYURY R&D AF2085 5 ug/ml
CLEAVED CASPASE BD Biosciences C92-605 4 ug/ml
CYCLIN B1 ThermoFisher GNS11 3 ug/ml
DNMT3a NovusBio 64B814.1 2ug/ml
DNMT3b NovusBio 52A1018 1 ug/ml
ESRRB R&D PP-H6705-00 1 ug/ml
ERK Cell Signaling Tech 137F5 4 ug/ml
pERK BD Biosciences 561991 5 ug/ml
GATA6 R&D AF1700 4 ug/ml
FOXA2 Santa Cruz 6554 1 ug/ml
HISTONE H3 Cell Signaling Tech 4499 2 ug/ml
pHISTONE H3 Abcam ab10543 0.001 ug/ml
KLF4 Abcam ab75486 2.5 ug/ml
KLF5 R&D AF3758 2 ug/ml
KI67 Cell Signaling Tech 9449 2 ug/ml
NANOG eBio MLC-51 2 ug/ml
OCT6 Millipore MABN738 2.5 ug/ml
OCT4 Santa Cruz sc8628 0.75 ug/ml
OTX2 R&D AF1979 1 ug/ml
pSMAD1/5 Invitrogen 700047 2 ug/ml
pRB Cell Signaling Tech 8516 2 ug/ml
SOX1 R&D AF3369 1.5 ug/ml
SMAD2/3 Cell Signaling Tech 8685 2.5 ug/ml
pSMAD2/3 Cell Signaling Tech 8828 5 ug/ml
SOX17 Neuromics GT15094 2 ug/ml
SOX2 Sigma-Aldrich ab5603 2.5 ug/ml
STAT3 Cell Signaling Tech 9139 5 ug/ml
TBX3 Santa Cruz sc17871 1.5 ug/ml
TFE3 Sigma-Aldrich HPA023881 2 ug/ml
TFCP2L1 R&D AF5726 5 ug/ml
ZNF281 Santa Cruz sc-166933 1.5 ug/ml
bCATENIN Cell Signaling Tech 8814 2.5 ug/ml
cMYC Cell Signaling Tech 5605 2.5 ug/ml
pmTOR Cell Signaling Tech 5536 5 ug/ml
php53 Cell Signaling Tech 9286 5 ug/ml
FOXA2_AF647 Abcam ab193879 1.25 ug/ml
NANOG_AF488 Ebioscience 53-5761-80 2.5 ug/ml
Donkey anti-goat AF555 ThermoFisher A-21432 2 ug/ml
FOXA2 Abcam ab40874 1:300 dilution
NKX2.2 DSHB 74.5A5, 1:100 dilution
Donkey anti-mouse IgG (H+L) Alexa FluorTM 488 Thermo Fisher A-21202 4 ug/ml
Donkey anti-mouse IgG (H+L) Alexa FluorTM plus 647 Thermo Fisher A32787 4 ug/ml
Donkey anti-rabbit IgG (H+L) Alexa FluorTM 488 Thermo Fisher A-21206 4 ug/ml
Donkey anti-rabbit IgG (H+L) Alexa FluorTM 546 Thermo Fisher A10040 4 ug/ml

Donkey anti-goat IgG (H+L) Alexa FluorTM 546 Thermo Fisher A-11056 4 ug/ml
, donkey anti-goat IgG (H+L) CF640R (Biotium, 20179,1:500).

Validation

AKT Cell Signaling Tech C67E7: Validated by company to work in Mouse and for immunohistochemistry
phAKT Cell Signaling Tech 4060: Validated by company to work in Mouse and for immunohistochemistry
BRACHYURY R&D AF2085: Validated by company to work in Mouse and for immunohistochemistry
CLEAVED CASPASE BD Biosciences C92-605: Validated by company to work in Mouse and for immunohistochemistry
CYCLIN B1 ThermoFisher GNS11: Validated by company to work in Mouse and for immunohistochemistry
DNMT3a NovusBio 64B814.1: Validated by company to work in Mouse and for immunohistochemistry
DNMT3b NovusBio 52A1018: Validated by company to work in Mouse and for immunohistochemistry
ESRRB R&D PP-H6705-00: Validated by company to work for immunohistochemistry. Validated by us as follows: dynamic expression range and nuclear localization in mouse embryonic stem cells during pluripotency and downregulation upon differentiation as expected.
ERK Cell Signaling Tech 137F5: Validated by company to work in Mouse and for immunohistochemistry
pERK BD Biosciences 561991: Validated by company to work in Mouse. Validated by us: dynamic expression range following immunofluorescence in mouse embryonic stem cells during pluripotency in SerumLIF as expected
GATA6 R&D AF1700: Validated by company to work in immunocytochemistry. Validated by us as follows: No expression in mouse embryonic cells in pluripotency, upregulation and nuclear localization upon meso-endoderm differentiation following immunofluorescence.
FOXA2 Santa Cruz 6554: Validated by company for mouse and immunocytochemistry
HISTONE H3 Cell Signaling Tech 4499: Validated by company for use in mouse and immunocytochemistry
pHISTONE H3 Abcam ab10543: Validated by company for use in immunocytochemistry and predicted to work in mouse. Published to work in mouse eg: Soffer A et al. Apoptosis and tissue thinning contribute to symmetric cell division in the developing mouse epidermis in a nonautonomous way. PLoS Biol 20:e3001756 (2022).
KLF4 Abcam ab75486: Validated by company to work in immunocytochemistry. Validated by us as follows: dynamic expression range and nuclear localization in mouse embryonic stem cells during pluripotency and downregulation upon differentiation as expected.
KLF5 R&D AF3758: Validated by company to work in immunocytochemistry and mouse.
KI67 Cell Signaling Tech 9449: Validated by company to work in immunocytochemistry. Validated by us as follows: dynamic expression range in mouse embryonic stem cells and upregulation during mitosis.
NANOG eBio MLC-51: Validated by company to work in Mouse and for immunocytochemistry
OCT6 Millipore MABN738: Validated by company to work in Mouse and for immunocytochemistry
OCT4 Santa Cruz sc8628: Validated by company to work in Mouse and for immunocytochemistry
OTX2 R&D AF1979: Validated by company to work for immunocytochemistry. Validated by us for immunofluorescence as follows: dynamic expression range and nuclear localization in mouse embryonic stem cells in pluripotency and upregulation during neural differentiation as expected
pSMAD1/5 Invitrogen 700047: Validated by company to work in Mouse and for immunocytochemistry
pRB Cell Signaling Tech 8516: Validated by company to work in Mouse and for immunocytochemistry
SOX1 R&D AF3369: Validated by company to work in Mouse and for immunocytochemistry
SMAD2/3 Cell Signaling Tech 8685: Validated by company to work in Mouse and for immunocytochemistry
pSMAD2/3 Cell Signaling Tech 8828: Validated by company to work in Mouse
SOX17 Neuromics GT15094: Validated by company to work for immunocytochemistry. Validated by us as follows: No expression in mouse embryonic cells in pluripotency, upregulation and nuclear localization upon meso-endoderm differentiation following immunofluorescence.
SOX2 Sigma-Aldrich ab5603: Validated by company to work in Mouse and for immunocytochemistry
STAT3 Cell Signaling Tech 9139: Validated by company to work in Mouse and for immunocytochemistry
TBX3 Santa Cruz sc17871: Validated by reviewers on company website to work in mouse and immunofluorescence for mESC. Same observed by us.
TFCP2L1 R&D AF5726: Validated by us for immunofluorescence as follows: dynamic expression range and nuclear localization in mouse embryonic stem cells during pluripotency and downregulation upon differentiation as expected.
ZNF281 Santa Cruz sc-166933: Validated by company to work in Mouse and for immunocytochemistry.
bCATENIN Cell Signaling Tech 8814: Validated by company to work in Mouse and for immunocytochemistry.
cMYC Cell Signaling Tech 5605: Validated by company to work in Mouse and for immunocytochemistry.
pmTOR Cell Signaling Tech 5536: Validated by company to work in Mouse and for immunocytochemistry.
php53 Cell Signaling Tech 9286: Validated by company to work in Mouse and for immunocytochemistry.
TFE3 Sigma-Aldrich HPA023881 : Validated by company to work in Mouse and for immunocytochemistry. Advanced validation done by RNAi knockdown.
FOXA2_AF647 Abcam ab193879: Validated by company to work for immunocytochemistry. Validated by us as follows: Correlates well with FOXA2mCherry reporter expression at single cell level.
NKX2.2 DSHB 74.5A5: Validated by company to work in Mouse and for immunocytochemistry.
NANOG_AF488 Ebioscience 53-5761-80: Validated by company to work in Mouse and for immunocytochemistry.
FOXA2 Abcam ab40874: Validated by company to work in Mouse and for immunocytochemistry

In addition, prior to creating the IMC panel, all antibodies were tested to be appropriate for mESCs via immunofluorescence. Antibodies were validated based on expression dynamic range, expected sub cellular localization and expected expression during pluripotency, differentiation, cell division etc based on literature.

Eukaryotic cell lines

Policy information about cell lines and Sex and Gender in Research

Cell line source(s)

R1 WT: Nagy, A., Rossant, J., Nagy, R., Abramow-Newerly, W. & Roder, J. C. Derivation of completely cell culture-derived mice from early-passage embryonic stem cells. Proc. Natl. Acad. Sci. U. S. A. 90, 8424–8428 (1993).
R1 NanogVenus: Filipczyk, A. et al. Network plasticity of pluripotency transcription factors in embryonic stem cells. Nat. Cell Biol. 17, 1235–1246 (2015).
NG4 (NANOG GFP, CCE line): Schaniel, C. et al. Smarcc1/Baf155 Couples Self-Renewal Gene Repression with Changes in

	Chromatin Structure in Mouse Embryonic Stem Cells. Stem Cells 27, 2979–2991 (2009). Sox1-EGFP (46C): Aubert, J. et al. Screening for mammalian neural genes via fluorescence-activated cell sorter purification of neural precursors from Sox1-gfp knock-in mice. Proc. Natl. Acad. Sci. 100, 11836–11841 (2003).
Authentication	Cell lines were not genetically authenticated
Mycoplasma contamination	Cell lines were tested for mycoplasma contamination and found to be negative
Commonly misidentified lines (See ICLAC register)	No commonly misidentified lines were used

Animals and other research organisms

Policy information about studies involving animals; ARRIVE guidelines recommended for reporting animal research, and Sex and Gender in Research

Laboratory animals	Animals used were 15- 20 weeks old RjOrl:SWISS mice from Janvier Labs
Wild animals	No wild animals were used
Reporting on sex	Sex was not considered in the design of the study experiment.
Field-collected samples	No samples were collected from field
Ethics oversight	All experiments were performed according to Swiss federal law and the institutional guidelines of ETH Zurich as well as approved by local animal ethics committee of Basel-Stadt (approval number 2655)

Note that full information on the approval of the study protocol must also be provided in the manuscript.

Flow Cytometry

Plots

Confirm that:

☒ The axis labels state the marker and fluorochrome used (e.g. CD4-FITC).

☒ The axis scales are clearly visible. Include numbers along axes only for bottom left plot of group (a 'group' is an analysis of identical markers).

☒ All plots are contour plots with outliers or pseudocolor plots.

☒ A numerical value for number of cells or percentage (with statistics) is provided.

Methodology

Sample preparation	R1WT or Sox1-EGFP/FoxA2mCherry cells were washed 3x with PBS post trypsinization, resuspended in PBS + 10% Serum before undergoing FACS analysis/sort. 100 um nozzle was used.
Instrument	BD FACS AriaIII
Software	BD FACS Diva v8 for data acquisition and FlowJo v10 for data analysis
Cell population abundance	Based on differentiation stage, 0-10% cells were Sox1EGFP+FOXA2mCherry-, 0-15% cells were Sox1-EGFP-FOXA2mCherry+ and 0-1% cells were Sox1-EGFP+FOXA2mCherry+. Downstream transcriptomic analysis validated our gating strategy as sorted populations displayed distinct lineage signatures.
Gating strategy	Gating strategy included commonly used gates for SSC/FSC for live cell isolation and doublet exclusion. Fluorescence gates were set based on R1WT negative control.

☒ Tick this box to confirm that a figure exemplifying the gating strategy is provided in the Supplementary Information.
